# DNA Microsystems for Biodiagnosis

**DOI:** 10.3390/mi11040445

**Published:** 2020-04-23

**Authors:** Alana Torres Vidal, Igor L. Medintz, Hieu Bui

**Affiliations:** 1 Department of Electrical Engineering and Computer Science, The Catholic University of America, Washington, DC 20064, USA; torresvidal@cua.edu; 2 Center for Bio/Molecular Science and Engineering, Code 6900, U.S. Naval Research Laboratory, Washington, DC 20375, USA; igor.medintz@nrl.navy.mil

**Keywords:** diagnosis, DNA nanotechnology, DNA-based medicine, nucleic acids

## Abstract

Researchers are continuously making progress towards diagnosis and treatment of numerous diseases. However, there are still major issues that are presenting many challenges for current medical diagnosis. On the other hand, DNA nanotechnology has evolved significantly over the last three decades and is highly interdisciplinary. With many potential technologies derived from the field, it is natural to begin exploring and incorporating its knowledge to develop DNA microsystems for biodiagnosis in order to help address current obstacles, such as disease detection and drug resistance. Here, current challenges in disease detection are presented along with standard methods for diagnosis. Then, a brief overview of DNA nanotechnology is introduced along with its main attractive features for constructing biodiagnostic microsystems. Lastly, suggested DNA-based microsystems are discussed through proof-of-concept demonstrations with improvement strategies for standard diagnostic approaches.

## 1. Introduction

Although researchers continuously achieve progress towards the diagnostics and treatment of numerous diseases, there are still major issues that challenge medical procedures and methods [1]. Emerging bionanotechnology, in particular DNA nanotechnology, can be incorporated in microsystems for biomedicine to help overcome current obstacles, such as disease detection. Among current medical challenges, misdiagnosis and drug resistance severely jeopardize treatments [2,3]. The former keeps the patient from receiving proper treatment in a timely fashion, which can aggravate the patient’s conditions, while the latter can be induced by misdiagnosis and have consequences that range from bacterial mutation—in case of infections—to damage of healthy tissue in oncological patients [3]. Point-of-care testing (POC) is a non-laboratory approach to providing immediate results to individual patients with high precision and accuracy. The goal of POC has evolved from prevention and early detection of disease to more personalized medicine through tailoring of interventions to individual patients. Recent improvements in the field of bionanotechnology can offer high specificity and accuracy in the development of personalized therapeutic strategies [4,5,6]. For example, bacterial resistance is a constant concern in the medical field because bacteria can mutate and spread easily, which reduces the efficiency of antibiotic medication. Thus, a thorough specification of the medication used to fight infections is vital to prevent epidemics and to keep patients from reinfection [3,7]. 

Applications of DNA nanotechnology for biomedicine aim to address the above-mentioned issues, such as reducing the time factor, misdiagnosis and drug resistance [8,9,10]. For instance, DNA de-methylation is related to the causes of autoimmune diseases—systemic lupus erythematosus, rheumatoid arthritis, systemic sclerosis, etc. and it can also be used as a biomarker of drug resistance [11]. In addition, the specificity of medication and treatment is another significant concern in the medical field, since it is crucial to targeting only the problematic cells while preserving healthy tissue and reducing side effects of treatments [12]. Certain medical conditions, however, need further attention. Idiopathic disorders, such as Alzheimer’s disease and autoimmune hepatitis, affect a large portion of society but still lack an accurate approach towards prevention, diagnosis, and treatment. Furthermore, current cancer therapies can bring a burden to the patient, mainly due to undesirable side-effects and unresponsive treatments. Here, current challenges in the detection of these conditions are briefly reviewed, as well as developing DNA-based mechanisms that could potentially address them. Diagnosis and treatment are equally applicable to and equally important in agriculture and veterinary medicine. Indeed, nanotechnology is applied to smart systems that self-regulate the release of antimicrobials and fertilizers in agriculture. Both end consumers and ecosystems are further protected when the control over chemicals used in agriculture is increased, and that is achieved through the high specificity and stability of nanostructures. For example, mesoporous silica nanoparticles (NPs) are used for the targeted delivery of stimulants and pesticides, while nanoscale biosensors such as those made from carbon nanomaterials are engineered for early detection of possible disease outbreaks, which enhances food safety [13]. Moreover, diagnostics is also improving in veterinary medicine by using quantum dots that can rapidly identify cancerous cells in animals [14,15]. In other words, applications of nanotechnology are of high importance to disease prevention and drug delivery in plants, animals, and humans. Although the present discussion is focused on the latter, it is important to highlight the versatility of smart and emerging DNA microsystems—groups of individual nucleic acid components that form a functional network with and without the existing diagnostic systems for achieving specific and augmented sensing and detecting purposes. In the following, we utilize several representative diseases to highlight the remaining challenges in both their diagnosis and treatment. Although not clearly encompassing all diseases, these are used since DNA technology and DNA-based materials have much to offer in terms of their diagnosis and treatment that are still unmet with current technologies.

## 2. Current Challenges in Disease Detection and Treatment 

### 2.1. Current Challenges in Alzheimer’s Disease

Alzheimer’s disease is a neurodegenerative condition that compromises the patient’s memory by causing neural and synaptic losses. Although Alzheimer’s pathogenesis affects millions of people—mostly at an advanced age—worldwide, it still does not have a cure nor an efficient way to be detected at an early stage [16,17]. With respect to the morphological changes in the human brain, there is a strong correlation in the different progressing phases of Alzheimer’s disease as a function of plaque development and the changes can be observed more pronounced via the CT scan in the latter stages [18]. Hence, the goal of nanotechnology-based therapies is to develop nanostructures and nanosystems to sense, for instance, the early formation of amyloid-beta (Aβ) plaques, especially monomers and oligomers, which are believed to be the primary causing agents of dementia in this case [19]. Related to this, activity-dependent neuroprotective protein (ADNP)-haploinsufficiency has also been linked with different responses to stress in mice, in a study that used pituitary adenylate cyclase-activating peptide (PACAP) as an ADNP regulator. Thus, ADNP-deficiency, among other concurrent disorders, is also associated with post-traumatic stress disorder (PTSD), Alzheimer’s disease, and schizophrenia [20]. Besides the difficult task of producing efficient biosensors for an early diagnosis, medical practitioners are also short on ways to decelerate and treat the condition. A promising approach is to employ RNA-based therapy, which uses double-stranded RNA to silence specific genes that could be related to the disorder [16,21]. Lastly, efficient drug-delivery also imposes a limitation to the treatments currently available. Therefore, nanocarriers need to have particular features, including but not limited to specificity, long blood circulating times, and low toxicity [19]. 

### 2.2. Current Challenges in Autoimmune Hepatitis (AIH)

Autoimmune disorders induce pathological responses from the immune system, which fail to make a distinction between self and foreign antigens. The overproduction of antibodies in the organism damages healthy cells [22]. As there are many variations of autoimmune diseases, the diagnosis is done based on a set of exam results, making it susceptible to misdiagnosis [11]. The detection of autoimmune hepatitis (AIH) is no longer an issue in the medical field because of the availability of reliable procedures used to diagnose other hepatic conditions [23]. However, an ideal detection procedure would not count on the exclusion method. For instance, the presence of antinuclear antibodies (ANA) does not indicate AIH, as other liver diseases can also trigger this autoantibody [23]. Moreover, healthy patients might also be seropositive, which challenges the use of ANA as a sufficient biomarker for autoimmunity; thus, the liver biopsy is often necessary to obtain a more comprehensive diagnosis of autoimmune hepatitis [24,25]. The standard treatment for autoimmune hepatitis is to administer medication such as prednisolone (PSL) or azathioprine to the patient. However, some patients might not respond well to the treatments mentioned, so the current alternative medication is mycophenolate mofetil, which unfortunately causes undesired side-effects [23]. For the reasons mentioned above, the utility of DNA nanotechnology applications would be beneficial to develop adequate and precise histological procedures to detect, monitor, and treat autoimmune diseases. For instance, DNA-based drug treatment can provide an alternative uptake pathway to minimize the side effects in patients which do not respond well to traditional treatment such as azathioprine or prednisolone. On the other hand, up-regulated biomarkers from liver due to autoimmune hepatitis can be targeted with programmable DNA constructs that can detect and amplify even if those biomarkers exist in a minute quantity. 

### 2.3. Current Challenges in Disease-Causing Mutations

In order to develop better methods to address Mendelian disorders, it is fundamental to know the cause and location of specific gene mutations; thus, the field of genomic medicine continuously aims to implement comprehensive exome and genome sequencing (WES and WGS), but there is still room for progress [26,27,28,29]. Current challenges include improving cost-effectiveness and exome selection, as well as making more specific diagnoses considering heterogeneity of disease variants and phenotypic similarity [30]. Furthermore, another disruption that delays proper identification of disease-causing mutations is the assortment of anomalies in genes, which includes but is not limited to single-base pair substitutions, inversions, and duplication [26]. For instance, inherited retinal dystrophy (IRD) presents a variety of phenotypes and is linked to more than 250 genes mutations, but the WES sequencing methods currently available for diagnosis are still limited [31]. Likewise, the identification of causes for inherited blindness remains uncertain as different genes were recently found to be concomitantly responsible for the development of the condition [32]. Also, with the challenge of identifying gene variants, genomic practices critically need to understand the importance of each gene mutation and interaction in the studied disorders [33,34]. Lastly, another concern is that some treatments are not yet efficient to address disease-causing mutations. Cystic fibrosis (CF), for example, is triggered by a mutation in the cystic fibrosis transmembrane regulator (CFTR) gene, which can be only potentially corrected by drug combinations—as opposed to a single therapy—to reach stabilization and retrieve functionality of the gene [35]. Recently, DNA-based X-probes were designed and demonstrated in vitro to detect rare single-nucleotide mutations with high specificity and sensitivity up to 3000-fold compared to their corresponding wild-type sequences [36]. It remains an exciting unexplored area in which next-generation DNA probes have the capability to augment existing methodology to assist clinicians determining treatment paths for advancing personalized medicine.

### 2.4. Current Challenges in Detection of Circulating Tumor Cells

**Figure 1:**
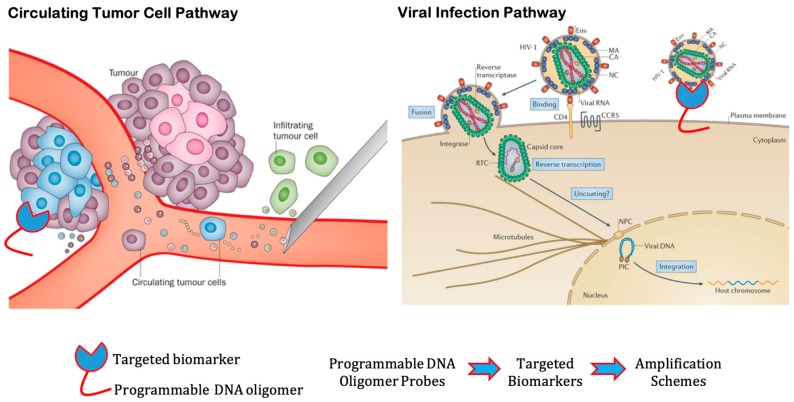
Current challenges in disease detection: (**Left panel**) Circulating tumor cells (CTCs) tend to be very low abundance and low affinity binding. Reagents are insufficient to finding and/or extracting those cells; (**Right panel**) Viral infections are highly specific and time consuming to construct prototypes on-demand. Hence, programmable DNA oligomers labeled with targeted biomarkers with high binding affinity can be exploited to detect and amplify the low abundance CTCs as well as detect and sense other relevant biomarkers such as viral particles. Adapted with permission from [39,45]. Copyright 2015, Springer Nature.

Cancer is one of the leading causes of death worldwide. Matured cancer cells without early detection may spread away from the primary tumor location and form metastases. The accurate analysis of circulating cancer cells (CTCs) can be used as a supplementary tool for screening and monitoring treatments, since it is less onerous than tissue biopsy [37]. In fact, variation in the number of circulating cells can indicate whether or not a patient suffering from prostate cancer is responding to radiation therapy [38]. A model of circulating tumor cells entering the bloodstream is depicted in Figure 1 (left panel) [39]. By analyzing CTC characteristics, artificial DNA probes can be programmed to intercept metastases or to probe the migration pathway within the bloodstream by functionalized oligomers with their corresponding surface-bound proteins. Unfortunately, CTCs have a different morphology and immunostaining pattern, even when they are originally from the same tissue [40]. Another prominent interest targeted is theranostics, a practice that combines diagnosis, monitoring and treatment on the same molecular scaffold in order to target specific cells [41]. Although theranostics is currently more commonly focused on cancer treatments, its methods could also be applied to other disorders [41]. The advantage of implementing a specialized treatment is minimizing the side-effects of chemotherapy or chemotherapeutics in patients by reducing the levels of toxicity due to the use of radioisotopes [12]. Moreover, DNA nanocarriers are employed in theranostics for drug-delivery in a “key-lock” mode, i.e., the nanostructures are designed with aptamers to match lock-like receptors displayed on tumor tissue only, leaving healthy cells unaffected [42]. Thus, the use of programmable nano-objects in theranostics avoids drug resistance and improves stability, specificity, and efficacy of drug-delivery systems in breast, liver, lung, and kidney cancer [42,43]. In fact, DNA cages have shown a promising performance—as opposed to DNA nanotubes and DNA plasmids—at drug-delivery and entrapment, due to their high surface area and strong interactions with specific compounds [43,44].

### 2.5. Current Challenges in Viral Infections

The time-lapse assessment between initial infection and the actual detection of specific antibodies, during which the patient is contagious, is among the ongoing hurdles of detecting viral diseases [46]. Indeed, the efficiency of treatment of RNA viral infections strongly depends on how soon the contamination is discovered; thus, point-of-care testing and prompt lab results are essential for reliable healthcare [47]. Another reason for prioritizing early detection is that not all viral diseases show symptoms soon upon infection, which delays detection and treatment [46,47]. A well-accepted model of reverse transcription in early HIV-1 viral replication cycle is depicted in Figure 1 (right panel) [45]. Using this model, DNA-based probes can be used to target relevant surface-bound proteins of viral particles (e.g., proteins associated with N gene or S gene), thus allowing clinicians to detect prior to the infection period. Upon hybridization to the targets, DNA probes can transduce their signals to the existing amplification schemes such as enzyme-based (PCR, ELISA, etc.) or DNA-enzyme-free (HCR, CHA, etc.). Highly sensitive tools are needed in order to obtain accurate screenings, especially for coexisting or small viral populations [48]. In myocarditis patients, currently employed methods for detection include magnetic resonance imaging (MRI) and endomyocardial biopsy (EMB) which, although promising, still lack sensitivity for direct pathogen detection [49]. Additionally, the relation between viral infections and autoimmune diseases is also a subject of study in medicine. For instance, patients with autoimmune hepatitis show a higher predisposition to viral hepatitis, such as HAV, HEV, HBV, and HCV, than the unaffected population [50].

## 3. Current Methods for Diagnosis

Current methods of diagnosis rely on the detection of DNA mutations in the patient or the DNA of the pathogen itself. Microscopic agglutination test (MAT) and enzyme-linked immunosorbent assay (ELISA) are popular methods that are currently implemented in diagnosis; however, live antigens are needed in the process for DNA matching, which not only increases cost but the chance of cross-reactivity. On the other hand, polymerase chain reaction (PCR) requires previous knowledge of the pathogen’s DNA sequence, so DNA inhibition and DNA replication errors may occur. Specificity, precision and speed are some of the factors that determine the cost-benefit of each procedure. Thus, medicine is constantly improving methods of diagnosis.

### 3.1. Microscopic Agglutination Test (MAT)

**Figure 2:**
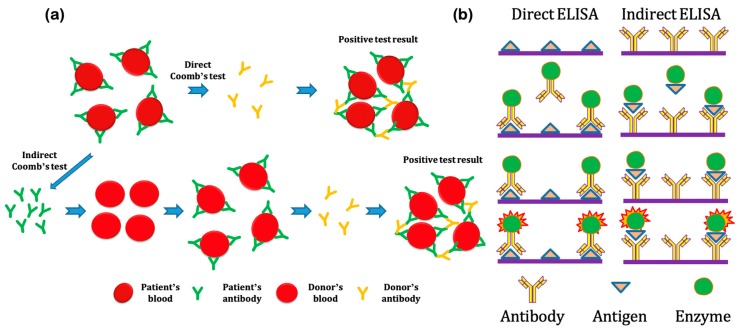
Current techniques to detect serological biomarkers: (**a**) Microscopic agglutination test (Coomb’s test) can be performed directly and indirectly. The direct test exposes the patient’s blood with the donor’s antibody whereas the indirect test exposes the patient’s antibody to the donor’s blood. In both cases, the positive results are associated with the formation of cell clumps; (**b**) Enzyme-linked immunosorbent assay (ELISA) can be performed directly and indirectly. The direct test binds the antigen of interest to an immobilized surface whereas the indirect test binds the antibody of interest to the immobilized surface. The positive results are associated with the color changes due to substrate and enzyme interaction.

The microscopic agglutination test (MAT) is commonly used for the diagnosis of bacterial infection [51]. MAT can be performed either directly or indirectly through the interaction between antibodies and blood cells. Figure 2a illustrates the principle of both direct and indirect agglutination tests, which are also called Coomb’s tests. The direct Coomb’s test detects surface-bound antibodies of blood cells whereas the indirect Coomb’s test detects antibodies that are free floating in the bloodstream and tend to interact against specific blood cells, both resulting in agglutination or a formation of cell clumps. Prenatal testing is an example of the indirect Coomb’s test whereas autoimmune hemolytic anemia testing is an example of the direct Coomb’s test. Although MAT is the reference serological test to detect agglutinating antibodies, it has limited sensitivity for early stages of infection, when compared to other immunodiagnostics [52]. New approaches to supplement or replace MAT are interesting to the medical field mainly because of cost-effectiveness, since MAT testing requires painstaking culture and manipulation of live antigens. Additionally, MAT cross-reactivity is common when used with different bacterial serotypes, which obstructs the identification of the infection etiology. For these reasons, more practical and accessible methods of diagnosis are being considered such as ELISA IgM, polymerase chain reaction (PCR), and counterimmunoelectrophoresis (CIE) [51,52]. 

### 3.2. Enzyme-Linked Immunosorbent Assay (ELISA)

ELISA, also known as enzyme immunoassays (EIA), is a serological practice for detection and estimation of antibodies and antigens [53,54]. Not only is it more stable than radioimmunoassays, ELISA also offers sensitivity and specificity by signal amplification [53]. In order to perform the analysis, antibodies are deposited on a support and they will later react to soluble antigens—a process that can be qualified and quantified by the color produced by an enzyme substrate added to the sample, as shown in Figure 2b. ELISA can be performed directly and indirectly. Direct ELISA first binds with an antibody to the antigen of interest to an immobilized surface. Then a customized antibody-enzyme complex hybridizes with the surface-bound antigen. Substrate and enzyme interaction create color change for signal detection and amplification. Likewise, indirect ELISA first binds the antibody of interest to an immobilized surface. A customized antigen-enzyme complex hybridizes with the surface-bound antibody. Similar to the signal detection and amplification as described in direct ELISA, the color change results from the substrate and enzyme interaction. For instance, commonly used substrates such as 3,3’,5,5’-tetramethylbenzidine (TMB) and o-phenylenediamine dihydrochloride (OPD) are used together with horseradish peroxidase enzyme (HRP) to create blue and amber colors, respectively, for signal detection and amplification [55]. In addition to direct and indirect ELISA’s, other variations of ELISA include competitive, indirect competitive, sandwich, and open sandwich ELISA (OS-ELISA) [56,57]. This analytical method shows advantages such as not being invasive—i.e., efficiency towards small amounts of tissue, and agent stability [58,59]. For cases of bacteria, ELISA (IgM-ELISA) performs better at initial phases of the disease, compared to MAT [60]. 

### 3.3. Polymerase Chain Reaction (PCR)

**Figure 3:**
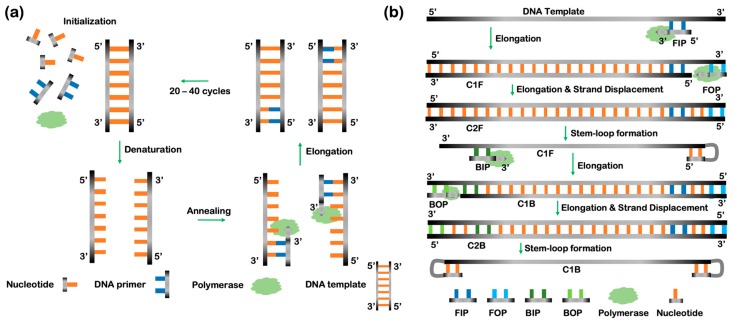
Enzyme-based techniques to detect disease biomarkers: (**a**) Polymerase chain reaction (PCR) uses polymerases, dNTPs, primers, and DNA template to synthesize exponential copies of the DNA template for signal detection and amplification via multiple thermal cycles; (**b**) Loop-mediated isothermal amplification (LAMP) extends the utility of PCR without requiring thermal cycling. Under isothermal conditions, four primers (FIP, FOP, BIP, BOP) sequentially hybridize to the DNA template while the polymerase simultaneously synthesizes C1F, C2F, C1B, and C2B copies of the template. The final stem-loop copy (C1B) is exponentially amplified for signal detection.

PCR is an assay that uses DNA polymerase to replicate a source DNA fragment in order to perform both quantitative (real-time PCR) and qualitative analysis (yes/no) of the sample [61,62]. The DNA fragment is often called a DNA template and typically exists in a small quantity and contains rich genetic information in order to probe a biological process including either the expression of a gene of interest or the identification of infectious agents. Using PCR, copies of the small DNA template are exponentially amplified through a series of thermal cycles. As illustrated in Figure 3a, the process of PCR typically consists of four steps (initialization, denaturation, annealing, and elongation). Prior to the initialization, a solution of DNA template, DNA primers (forward and reverse primers), deoxyribonucleotide triphosphates (dNTPs), and polymerase is mixed together. After the polymerase is thermally activated (> 90 °C) in the initialization step, the denaturation step heats the solution and causes DNA melting, breaking the dsDNA template into two single stranded (ss) DNA halves. The primers hybridize to the ssDNA templates during the annealing step. The polymerase then synthesizes a new DNA strand complementary to the ssDNA templates by adding free dNTPs from the reaction mixture in the elongation step. This step is repeated for multiple cycles, resulting in exponential copies of the DNA template. More advanced PCR techniques have been developed and reported extensively elsewhere [62,63,64,65,66]. For example, real-time PCR can monitor the amplification of a template in real time via fluorescence-labeled probes whereas digital PCR can measure the quantity of a template being synthesized in an encapsulated droplet. In general, advanced PCR techniques often require the use of expensive propriety equipment and fluorescence-labeled probes for either quantitative or qualitative analysis, thus increasing the cost of assay development. In addition, it remains difficult to deploy advanced PCRs in remote areas and those techniques are more prone to error with inexperience users. While PCR has advantages such as being simple to implement and highly sensitive, one of its limitations is only being able to identify pathogens that are previously known [63]. Compared to MAT, PCR is a faster, less complex alternative for the detection of bacteria because it does not require cultures [64]. More precisely, a combination of PCR and IgM-ELISA can quickly diagnose the disease at very early stages. This analytical method is often applied in several fields, such as virology and parasitology, as part of the diagnosis procedure [64]. In oncology, quantitative PCR can be used to detect circulating cancer cells and improve prognosis [65,66]. However, PCR is susceptible to unpredictable inhibitions, which can obstruct detection; thus, inhibitor detectors and controls are needed [67]. Although PCR is faster, inexpensive, highly sensitive, and a mostly accurate method, there can be replication errors of the source DNA, which could later affect the remaining results.

### 3.4. Multiplex Methods for Diagnosis

Multiplex polymerase chain reaction (MPCR) is an optimization of the conventional PCR method; it uses multiple target sequences and multiple primers, in order to enhance the annealing rate of the primers and reduce reaction failures [68,69,70]. The benefits of the MPCR include better indications of the sample’s quantity and quality, along with a lower expense for implementation [70]. However, by increasing the amount of primers, the sensibility and specificity of the method can be compromised, due to higher chances of amplification errors and cross-reactions [68]. In order to overcome these limitations, chemically modified primers such as those incorporating 4-oxo-1-pentyl (OXP) can be implemented to avoid undesired amplification, thereby improving multiplex PCR [71]. Similarly to PCR, MPCR is used for rapid accurate diagnosis. Applications of MPCR include the diagnosis of tuberculosis [72], subtypes of influenza virus [73], bacterial pathogens in ducks [74], protozoan pathogens in oyster samples [75], as well as the detection of *Escherichia coli* (*E. coli*) and the presence of other bacteria in milk [76]. 

### 3.5. Loop-Mediated Isothermal Amplification (LAMP) 

LAMP uses a DNA polymerase, two inner primers and two outer primers to amplify a DNA template under constant temperatures [77]. A mixture containing the target DNA and four primers is heat denatured and rapidly cooled on ice. The LAMP reaction is then initiated by the addition of the *Bst* DNA polymerase and carried out at constant temperature (i.e., 65 °C) to synthesize many copies of DNA template, unlike the PCR method which cycles through a range of temperatures during the elongation step. The mechanism is highlighted in Figure 3b. Initially, the inner forward primer (FIP) hybridizes to the DNA template and synthesizes a new copy (C1F) of the DNA template. The outer forward primer (FOP) then hybridizes to the outer domain of the DNA template and synthesizes a new copy (C2F) of the DNA template while simultaneously displacing C1F via the strand displacement process. The C1F and C2F copies are almost identical except the latter copy has an additional domain. The inner backward primer (BIP) then hybridizes to C1F and synthesizes a new copy (C1B) of the DNA template. Undergoing a similar strand displacement mechanism, the outer backward primer (BOP) hybridizes to the outer domain of the C1B and synthesizes a new copy (C2B) of the DNA template. Similarly, the C1B and C2B copies are almost identical except the latter copy also has an additional domain. As expected, the C2B is self-assembled and forms a stem-loop DNA that is used as the starting material for the cycling step, which is the second stage of the LAMP reaction. For the cycling amplification stage, only FIP and BIP primers are involved to further synthesize the DNA template for signal detection. Although the LAMP method appears to be similar to the PCR method, there are at least three factors that allows the LAMP method to be distinct. The first factor is the use of secondary structures of the template (e.g., hairpin’s loop) [78,79]. The second factor is the use of polymerase-assisted strand displacement. The third factor is the use of several primers to achieve isothermal amplification. LAMP can be conventional and commonly used for diagnosis of human pathogens or implemented in formats including reverse-transcription, multiplex, and others [80]. LAMP uses auto-cycling strand displacement DNA synthesis, and it depends on the size of the target DNA [77]. Additionally, loop-mediated isothermal amplification methods can surpass PCR amplifications in terms of amplification failure, sensitivity and efficiency [77]. Since this analytical method is still mainly used in research laboratories, new approaches are being investigated to make it practical, such as developing a portable detection prototype based on Arduino, an open electronics micro controller board [81]. Currently, LAMP is more accurate than quantitative-PCR for the detection of meningococcal infections in children [82], gene doping therapies [83], peanut allergens in processed food [84], acute viral necrobiotic infections in scallops [85], and *Staphylococcus pseudintermedius* bacterial infections in canines [86]. 

### 3.6. Next-Generation Sequencing (NGS)

Next-generation sequencing (NGS) relates to a group of various parallel DNA sequencing techniques, which are more efficient replacements to classical Sanger sequencing as applied in gels or capillaries and do not require previously known target sequences [87,88]. Noteworthy techniques include applied biosystems sequencing, sequencing by hybridization, sequencing by synthesis, ion torrent sequencing, single-molecule-real-time sequencing (SMRT), etc. [89]. NGS can be used to discover new gene mutations, as well as qualitatively identify pathogens and possible interactions between them, and contribute to comprehensive diagnosis of cancer, rare Mendelian disorders, and immunodeficiencies, as the whole genome can be analyzed [87,90,91]. For the diagnosis of viral infections, NGS is convenient because it does not require specific reagents [92]. However, since the setup of the method is significantly more expensive than Sanger technology, NGS is advantageous for higher throughput and more complex sequencing analysis that needs to be done in a timely fashion [87,89]. A drawback of using NGS is that it often needs amplification prior to applying the actual sequencing process [93] and this challenge may benefit from integrating the newly reported X-probes from the field of dynamic DNA nanotechnology [36]. Moreover, the infrastructure needed to support large amounts of data for NGS is one of the main challenges of the technique [92].

## 4. Overview DNA Nanotechnology

DNA nanotechnology is a growing field that aims to program DNA and other related nucleic acid strands into desired patterns to form systems with potential applications that range from new methods of drug-delivery to digital information storage [94,95]. DNA nanotechnology is a bottom-up assembly approach that takes advantage of the complementarity of DNA strands and the fact that their axis are unbranched to build designer DNA 2D and 3D objects, arrays, nanowires, and other nanostructures [96]. DNA nanotechnology can be perceived as being constituted by two major areas: structural DNA and dynamic DNA [9,97]. More focused reviews on DNA nanotechnology have been reported extensively elsewhere [94,96,98]. In the following, we briefly describe several representative DNA architectures and their primary functional characteristics.

### 4.1. Structural DNA Nanotechnology 

**Figure 4:**
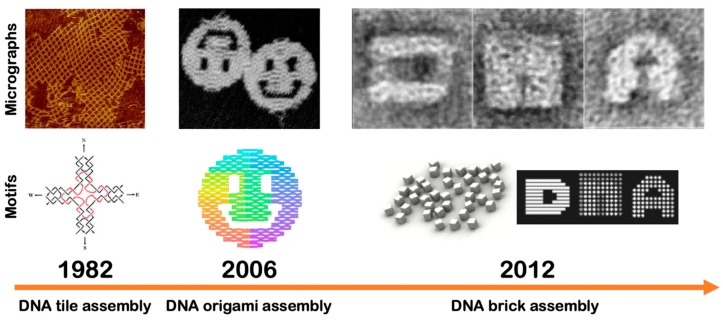
Three fundamental DNA self-assembly approaches for the design of precise DNA nanostructures are DNA tile assembly, DNA origami assembly, and DNA brick assembly with respect to the historical perspective. A representative micrograph of DNA lattices which are made from self-assembled distinct individual DNA tiles whereas DNA origami smiley faces are made from several hundred self-assembled short single-stranded DNA oligomers with a 7-kilobase scaffolded DNA strand. A micrograph of 3D DNA bricks which are made from the same 3D canvas, spells out the word “DNA”. Adapted with permission from [105]. Copyright 2003, DNA9. Adapted with permission from [106]. Copyright 2006, Springer Nature. Adapted with permission from [107]. Copyright 2012, Science.

Structural DNA nanotechnology aims to build artificial nanoscale objects made of DNA oligomers, with highly controllable shapes and sizes. The most common building blocks used in nanostructures are DNA tile assembly, DNA origami assembly, DNA brick assembly, and others [94] as illustrated in Figure 4. The strands are connected through sticky-ends and form optimized branched junctions that can be used as building blocks in nanostructures [99]. An immediate utility of DNA nanostructures has been employed in designing drug delivery vehicles with customized shapes and sizes to accommodate various drug molecules [100,101]. Moreover, DNA nanostructures typically use toehold strand displacement, which is based on hybridization and complementarity of strands, and this makes for dynamic structures, i.e., shape-shifting nanostructures [97]. The use of dynamic DNA systems has been explored in probe designs for sensing, detecting, and amplifying biomarkers such as RNA, proteins, etc. [102,103,104]. 

#### 4.1.1. Active and Passive DNA Nanostructures 

Active and passive DNA nanostructures are often programed based on Watson–Crick hybridization. One of the first demonstrations of passive DNA nanostructures was reported by Seeman et al. in the early 1980s [108]. They created the first DNA junctions which were inspired from the Holliday junction found in the transcription process. One of the first demonstrations of active DNA nanostructures was reported by Yurke et al. in early 2000s [109,110] via the construction of molecular mechanical tweezers. They successfully created DNA tweezers that can open and close in response to the addition of fuel strands. The mechanical opening and closing processes were successfully demonstrated via multiple cycles. 

#### 4.1.2. DNA Tiles 

DNA tiles are a subset of DNA nanostructures and were first demonstrated in the early 1980s [108]. The structures are made from programming several individual single-stranded DNA sequences to hybridize and form pre-determined structures. [111]. Many DNA tiles can be programmed to form DNA lattices by using crossovers such as the double-crossover (DX) and triple-crossover (TX), which provide rigidity and stability to the design of interest [94]. However, the challenges of using DNA tile assembly to build complex structures include the lack of control over the size, along with the requirement for new sequences at every new step [97]. 

#### 4.1.3. DNA Origamis 

DNA origamis are a subset of DNA nanostructures and were first demonstrated in 2006 [106]. The structures are programmed based on the prior sequence information of one long ssDNA scaffold combined with several hundred reverse short staples strands and complementary oligomers, in order to form specific designs [107,112]. The staple strands drive the folding of the scaffold by cross-linking it into the desired structures in a site-specific and addressable manner. The advantage of using a self-folding long scaffold is the control over stoichiometry where excess staples can be added to drive the reaction to completion [113]. In fact, the short-staples-scaffold/origami assembly approach invented by Paul Rothemund has a faster synthesis with high yield and does not depend on equimolar stoichiometry, as opposed to the traditional systemic design of oligonucleotides, but there may be missing strands in the final origami object [114]. As 2D and 3D nanostructures have been developed over the years, the DNA origami technique remains fundamental for the design of complex but precise DNA nanostructures [115]. 

#### 4.1.4. DNA Bricks 

DNA bricks are a subset of DNA nanostructures and were first demonstrated in 2012 [107,116]. The structures are programmed using many individual oligomers, single strands, in order to make a universal molecular canvas. Depending on the final size and shape, oligomers can be added or removed, which characterizes robustness. Thus, DNA bricks are modular units made from binding short synthetic strands—self-assembly—to be used in complex 3D nanostructures. Moreover, DNA bricks can be made from a range of motifs and patterns, according to the desired final shape, with perpendicular bindings. Recently, bricks were designed without binding domains and with more complexity, which further optimizes functionality and assembly [117]. 

### 4.2. Dynamic DNA Systems 

**Figure 5:**
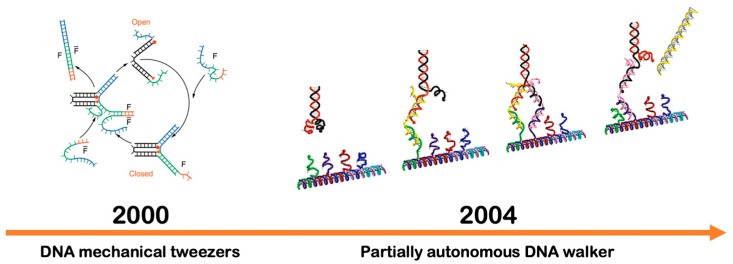
Dynamic DNA systems with capabilities to exhibit complex and controllable motions at the molecular scale. A schematic representation of programmable DNA tweezers shows its operations that can open and close repeatedly. Adopted with permission from [110]. Copyright 2000, Springer Nature. Utilizing the cascade DNA hybridization reaction, a programmable DNA walker can traverse along a predetermined DNA track. Adapted with permission from [120]. Copyright 2004, American Chemical Society.

Dynamic DNA systems are programmable structures that display partial and/or full autonomy once self-assembled together as illustrated in Figure 5. Unlike DNA nanostructures as described previously, the principle of most dynamic DNA systems is based on the exploitation of the Watson–Crick hybridization reaction and the strand displacement mechanism. For instance, a double-stranded DNA duplex with an overhang at the 5’/3’ end is used as the basis. Multiple individual bases are then programmed in a cascade reaction for achieving linear forward motions [118]. In general, the field of dynamic DNA nanotechnology aims to design systems that are often inspired by the behavior of molecular protein motors. In particular, DNA walkers are an example of a cascade reaction which translates multiple DNA hybridization reactions into a successive step-by-step linear motion [78,79,102,119]. These structures can potentially be used for transporting functional molecules or for designing active molecular transport and autonomous DNA nanomachines [97,120].

#### DNA Strand Displacement 

DNA strand displacement is a precise mechanism to program DNA oligomers to exhibit dynamic behaviors such as binding and unbinding for reusable purposes. It consists of the manipulation of toeholds (unpairing nucleotides) that control the hybridization process of complementary single-strands, in order to reconfigure input and output strands [97,121,122]. Most of the dynamic and active DNA nanostructures utilize the strand displacement process to achieve desirable motions at the molecular scale at low cost and high efficiency. As the complexity goes up, the propensity of crosstalk among active systems increases. Thus, it is imperative to design complex systems with the aids of computer tools such as NUPACK or Visual DSD to balance the Gibbs free energy [123,124]. 

### 4.3. Major Advantages and Limitations

As DNA nanotechnology grows as a field of study, more complex structures can be built using this bottom-up approach. Advantages include self-assembly, stability of DNA molecules, predictability of structures, and precision programmability at a low price [125]. Therefore, DNA nanotechnology may potentially be used even in oncology as structures for targeted drug-delivery and for biosensors with diagnostic capabilities [126,127]. Additionally, energy transfer systems and photonics, an emerging field for controlling electromagnetic waves at the molecular scale, can benefit from DNA nanometer-scale for energy production and harvesting [9,128,129,130]. However, there are limiting factors to the advances of DNA nanotechnology, including the availability of software designing tools that can increase the complexity and stability of nanostructures [113]. Furthermore, scalability is another current issue in the field, since it is necessary to implement practical functionalities in order to make the structures more advantageous [125]. Likewise, reliable sequence design and oligonucleotide synthesis are fundamental for the development of functional DNA structures [114,118]. 

### 4.4. Current DNA-Based Approaches Being Developed towards Diagnosis

DNA is biocompatible and an inexpensive material (~10 cents per nucleotide). DNA nanostructures are highly programmable and versatile units, which makes them relevant to next-generation biomaterials. DNA nanostructures can be cost-effective alternatives to existing diagnosis techniques and any functional molecule is readily available to couple with any DNA oligomer [131,132]. Additionally, DNA aptamers and DNAzymes are special molecules, generated via the systematic evolution of ligands by exponential enrichment (SELEX) process, that can bind to a vast number of different molecules [133]; they can be employed in various applications of nanotechnology, ranging from nanoelectronics to drug delivery and diagnosis [134]. In fact, targeted drug delivery and diagnosis are some of the most explored uses of nanotechnology. For instance, DNAzymes, aptamers, and gold NPs (AuNPs) are being developed for highly sensitive sensors that efficiently detect small molecules such as mercury (Hg^+^), which is extremely toxic to humans [135]. Also, AuNPs and photothermal detection are used in the diagnosis of specific bacteria [136]. Furthermore, electrochemical DNA sensors offer a faster, more specific, sensitive, and versatile detection modality for target pathogen molecules [137]. Moreover, nanocarriers can bind to commonly used drugs such as doxorubicin to reduce side effects, toxicity, and drug resistance in cancer therapy [138,139,140,141]. Nanorobots can be used to carry thrombin and stop blood flow in tumor vessels, while DNA-based signal amplification methods combined with DNA-integrated gold nanomaterials accurately detect tumor biomarkers [138]. Alzheimer’s disease biomarkers can also be detected using nanotechnology both in vitro and in vivo [142]. Detecting small molecules, bacteria, pathogen biomarkers as well as targeted drugs may be enhanced by utilizing the knowledge from the field of DNA nanotechnology in the near future due to the advantages described above.

## 5. DNA Biosensors

Identifying relevant genetic biomarkers can contribute to both prevention and subsequent monitoring of the response to a treatment [11]. In theory, DNA biosensors are nanodevices with high sensitivity and versatility. Biosensors can be designed to respond to targeted markers and can provide output for detection as electrical, chemical, optical signal or other modalities. Thus, biosensors must consist of a sensor component, a signal transducer, and a signal processor [143,144,145]. In addition, biosensors must have adjustable properties and a high ratio of surface area to volume [126]. The following subsections highlight the latest demonstrations of such DNA-based devices in design and/or in practice. 

### 5.1. DNA Nanostructures for Biomarker Diagnosis

**Figure 6:**
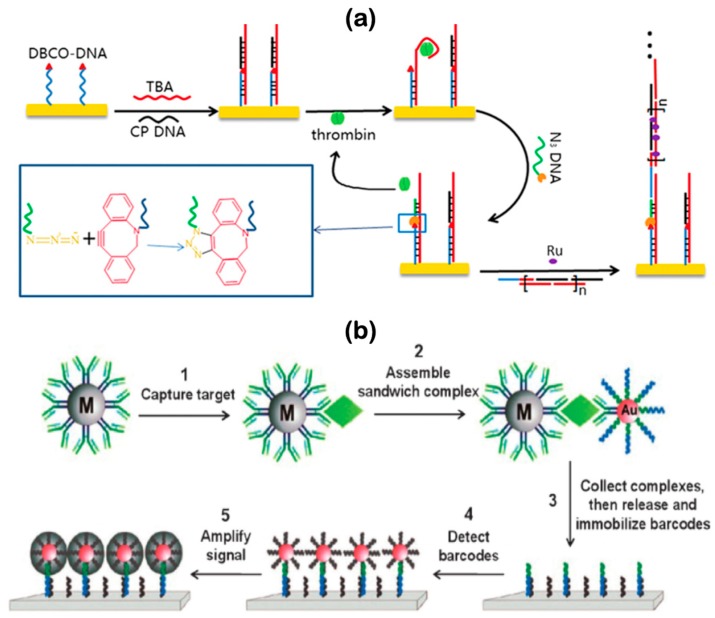
Applied DNA nanotechnology for the detection of nucleic acid and protein targets without PCR: (**a**) An electrochemical biosensor based on dual amplification to detect targeted thrombin as low as 30 fM; (**b**) A barcode microarray biosensor based on a combination of NPs and sequence-specific DNA with multiplexing different targeted molecules. Adopted with permission from [151]. Copyright 2015, Elsevier. Adopted with permission from [154]. Copyright 2011, The Royal Society of Chemistry.

Biosensors can either be bioaffinity, biocatalytic or microbe devices, which can bind to the target or use an immobilized enzyme for recognition, respectively [146,147,148,149]. Thus, biosensors can be implemented in ‘lab-on-a-chip’ devices, which are based on the integration of diagnostic processes [150]. Fundamental features that biosensors need to provide are selectivity, sensitivity, linearity of response, signal reproducibility, response and recovery promptness, and stability [129]. While biosensors offer high sensitivity and speed, current designs still lack perfect integration, automation, and simplicity (when compared to traditional methods) [143]. The following examples demonstrate the use of DNA nanostructures as an active component for proof-of-concept diagnosis. For instance, a recent enzyme-free electrochemical biosensor (Figure 6a) combined two DNA nanotechnology’s techniques i.e., DNA strand displacement and hybridization chain reaction (HCR), together with a conventional electrochemical amplification method to detect targeted thrombin, an important molecule in thrombosis and hemostasis [151]. In particular, the first amplification occurs when the target sequence (i.e., TBA) attaches to the protein and releases recognizable outputs (i.e., CP DNA and N3-DNA), which eventually releases the protein for a new reaction cycle. Moreover, the second amplification occurs with HCR, when the initiator (i.e., RP DNA) is added to hairpins and creates the secondary output signal in the form of dsDNA which acts as hexaammineruthenium chloride’s absorbent materials for producing amplified electrochemical signal. Thus, this assay allows highly sensitive detection of biomarkers with a limit of detection (LOD) of 30 fM. In addition, the assay offers high selectivity toward the target protein due to the specificity of the DNA aptamer and, without using active enzymes in the detection, the assay development’s cost is reduced [151]. By simply utilizing the DNA strand displacement technique, another proof-of-concept biosensor was capable of organizing proteins with highly diverse and complex logic processes for constructing synthetic protein switches. In particular, Boolean logic circuits were designed for sensing low-level cancer-specific mini-RNAs using DNA strand displacement via a series of controllable cascade enzyme reactions and the positive signal was then amplified for signal detection. Initially, input strands added to the mixture started the displacement of inactive DNA-complexes by freeing their toeholds, which were then triggered to interact with the remaining reactions. The system activity was measured by Förster resonant energy transfer (FRET), which provides minimal perturbation to the sample integrity with high photonic sensitivity [152]. Therefore, mini-RNAs can be inputs to DNA-circuits that detect the presence of cancer cells and can be further implemented in systems that release drugs to targeted cells [153]. The third demonstration of proof-of-concept biosensors is illustrated in Figure 6b, showing a schematic for protein detection based on DNA bio-barcode sensors. To do so, magnetic microparticles (MMPs) and AuNPs are encoded to match the target molecule. A DNA microarray chip is then hybridized with the barcodes, and the signal is amplified for better sensitivity [154]. Although it remains difficult to incorporate all the fundamental features of an ideal biosensor in practice, these results point to a promising trend for next-generation biosensors that ultimately can be operated on-site and on-demand. 

### 5.2. Hybrid Enzyme-DNA Biosensors

**Figure 7:**
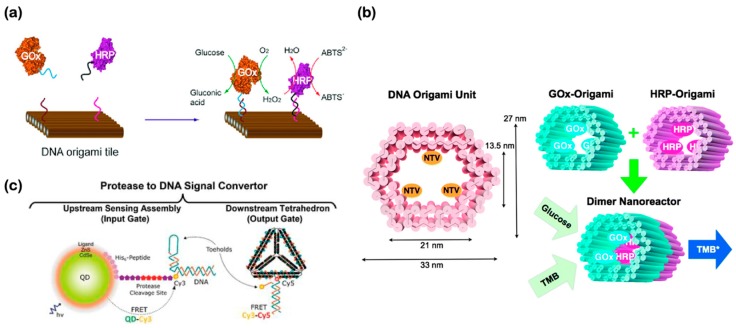
Hybrid enzyme-DNA biosensors: (**a**) DNA origami tile combined with programmable DNA sequences acts as a platform to control the spatial arrangement of cascade enzymatic activities. For instance, the reaction rate between GOx and HRP is tunable by adjusting the staple strands of the DNA origami tile; (**b**) A reactor-like system utilizes 3D DNA origamis to enhance enzymatic activities as a new confined space synthesis. In particular, GOx and HRP are internalized inside the interior of DNA origamis to prevent unintended reactions until the target is present; (**c**) Coupling DNA nanostructure and programmable DNA sequences to semiconductor quantum dots and peptides to construct a hybrid protease-to-DNA signal converter. Upon sensing the presence of target protease, the system converts a protease-peptide reaction into subsequent DNA hybridization reactions. Adopted with permission from [155]. Copyright 2012, American Chemical Society. Adopted with permission from [158]. Copyright 2015, The Royal Society of Chemistry. Adopted with permission from [145]. Copyright 2019, Wiley & Sons.

Enzymatic biosensors are commonly used in the food industry to identify pathogens, contaminants, and toxins such as bacteria and pesticides [149]. The biocatalytic nanodevices are based on co-immobilized enzymes that interact with the biological element of interest; however, such a setup can be complex and expensive [129]. Utilizing the flexibility and programmability of DNA materials, next-generation hybrid enzyme-DNA biosensors can offer inexpensive and highly modular constructs (Figure 7). For example, Figure 7a shows a proof-of-concept cascade enzymatic activity can be spatially controlled when combined with DNA nanostructures. The distance dependency of enzymes glucose oxidase (GOx) and horseradish peroxidase (HRP) is based on the spatial arrangement of the staples strands of the DNA origami rectangular. Those DNA staple strands offer a dual connectivity such that one end hybridizes to the DNA nanostructure and the other end hybridizes to the enzyme-specific strand of interest. Notwithstanding, the diffusion of enzyme-labeled DNA sequences (i.e., GOx and HRP) to the origami rectangular is also important for the reaction to happen [155]. In theory, the enzymatic activity here is expected to occur faster compared to that activity without the presence of the DNA nanostructure due to the dimensional reduction and the distance controllability [156,157]. By exploiting the 3D DNA nanostructures, the encapsulation of GOx and HRP into DNA origami modules coated with neutravidins (NTVs) and biotin demonstrates another dimension and versatility of DNA nanotechnology which effectively enhances the specific binding among targeted enzymes in a reactor-like system (Figure 7b). Since the resulting programmable DNA nanostructure is compartmentalized, the reaction activity is increased; thus, the nanostructure can be used as a promising diagnostic tool [158]. Not only are DNA nanostructures compatible and remain functional with the existing enzymes and other molecular molecules, DNA nanostructures are also compatible and remain functional with inorganic materials as well as peptides. For instance, a proof-of-concept system for the transduction of protease enzymes into DNA output has recently been demonstrated (Figure 7c). The input gate of the sensor is composed of quantum dots (QDs) enclosed in peptide-DNA (PD) and its peptide-toehold, which is recognized by the targeted enzyme. On the other hand, the output of the sensor is a tetrahedral DNA nanostructure, also with a toehold for hybridization. The activity is measured by FRET, which is reduced when the DNA output is released from the surface in the input gate. In other words, protease activity is converted into a DNA output and it is made available through specific enzyme activation (proteolysis) [145]. This converts what is essentially a single step biological process into an output that can interact with a DNA device and provide access to all that such technology affords. As illustrated in the above demonstrations, DNA nanotechnology can offer much more than what has been described in terms of structural architecture, modularity, and programmability for next-generation cell-free enzymatic detection. 

### 5.3. Hybrid DNA Electrochemical Biosensors

**Figure 8:**
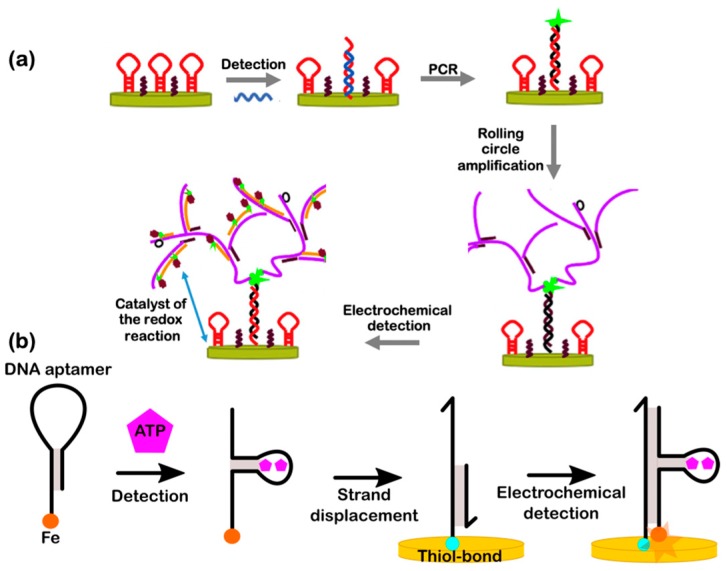
Hybrid DNA electrochemical biosensors: (**a**) By integrating programmable DNA sequences and active DNA systems on an existing electrochemical platform to detect biomarkers of interest by employing circular strand displacement polymerization and hybridization chain reaction amplification schemes; (**b**) Programmable DNA aptamers are immobilized on an electrochemical platform to sense the presence of ATP. Upon sensing the target molecule, the aptamer changes its structural conformation and hybridizes to the probe on the electrochemical surface, resulting in changes in electrochemical potential for signal detection. Adapted with permission from [160]. Copyright 2016, Elsevier.

Changes in electrochemical parameters can be captured by a high-sensitivity redox indicator [150]. Electrochemical detection is also based on electrical transduction, making it less complex than labeling detection and with higher biocompatibility towards other nanomaterials [143]. Such biosensors can use nucleic-acid hybridization, making it a competitive alternative to slower biosensors because its core components are electrodes with high signal-to-noise ratio (SNR), and they are culture-independent processes [159]. Unlike traditional approaches, hybrid DNA electrochemical biosensors have been explored recently utilizing highly programmable DNA sequences with the flexibility to target various biomarkers of interest (Figure 8). For instance, Figure 8a shows the schematic of an electrochemical biosensor based on circular strand displacement polymerization (CSDP) and hyperbranched rolling circle amplification (HRCA). The target DNA is detected by a molecular beacon (MB) hairpin-shaped probe, as several polymerization cycles of DNA strands occur. Then, the double-stranded DNA (ds-DNA) is created on the electrode surface by HRCA, which makes the biosensor readings highly sensitive because of its cascade operation [160]. Ultimately, the target DNA can be conjugated with other biomarkers such as RNA, proteins, peptides, enzymes, or other molecules, thus creating a massive library of sensor and barcoded recognition elements for multiplexed biosensing. Under optimal conditions, the reported biosensor has high sensitivity with a LOD as low as 8.9 aM. Likewise, Figure 8b shows the design of an electrochemical biosensor based on hairpin DNA aptamers, special secondary constructs often discovered via SELEX. The detection of adenosine triphosphate (ATP) is made by measuring the change in DNA strand aptamers. Thus, in the presence of ATP, toehold-mediated strand displacement will occur, and the activity will be read as an electrochemical signal [161].

## 6. DNA-based Medicine

Drug design and personalized therapy are the latest approach to combat difficult diseases such as cancers. DNA nanotechnology offers programmable DNA sequences with predictable structures which can be used in improving a drug’s uptake efficiency and targeting unhealthy cells in the vast space of physiological diversity. The following sections highlight a few representative proof-of-concept demonstrations which involved DNA materials for improving the drug delivery vehicle as well as personalized therapy.

### 6.1. DNA Nanostructures as an In Vivo Drug Delivery Vehicle

**Figure 9:**
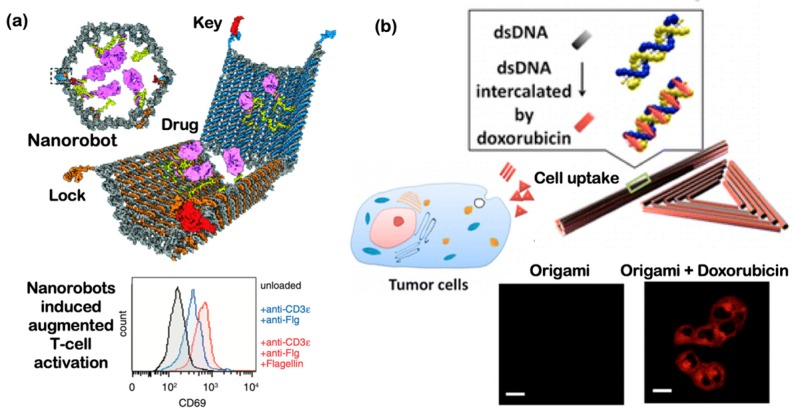
DNA-base nanomedicine: (**a**) Targeted drugs are sequestered within the interior of 3D DNA nanorobot with a controllable key-lock pair for specialized delivery purpose. Nanorobots loaded with combinations of antibody fragments are used in different types of cell-signaling stimulation (T-cell activation) in tissue culture; adapted with permission from [167]. Copyright 2012, Science. (**b**) Doxorubicin-loaded DNA nanostructures as an emerging delivery vehicle. Origamis with doxorubicin are successfully taken up by the cells as visualized via confocal fluorescent analyses. Adapted with permission from [166]. Copyright 2015, American Chemical Society.

The properties and characteristics of NPs used in drug delivery vary according to their use; thus, a meticulous matching between NP and drug incorporation is fundamental for an efficient drug delivery system [162]. For in vivo applications, characteristics like serum stability, cell uptake efficiency, target specificity and macrophage evasion are imperative [163]. The development of in vivo drug delivery vehicles aims to control drug release with ‘on’ and ‘off’ phases, targeting only specific cells and reducing toxicity in patients [164]. In fact, in vivo monitoring can be crucial to avoid Aβ peptide agglomeration, which is said to be the causing factor of neurodegenerative conditions such as Alzheimer’s disease [165]. As previously mentioned, nanoscale reactors based on DNA origami modules can be used in ultrasensitive assays for specific drug delivery, as DNA is highly programmable and biocompatible (Figure 9). For example, proof-of-concept drug carrier was made of DNA origami capsule in which target drugs were incorporated as illustrated in Figure 9a [158]. The capsule protected the drugs during the transport and upon sensing a target key, the capsule’s lock was released and the drugs were presented. Indeed, different lattices can be used to compose three-dimensional origami structures, and this can be achieved through the repetitive manipulation of programmable DNA scaffolds crossovers between helices and staple strands [100]. Another demonstration of using various DNA nanostructures for drug delivery vehicles to study the uptake capability through various geometrical shapes is illustrated in Figure 9b. A triangular DNA origami and a nanotubular DNA origami were loaded with doxorubicin, a chemotherapy medicine used to treat breast cancer, AIDS-related Kaposi’s sarcoma, ovarian cancer, bladder cancer, small cell lung cancer, and multiple myeloma [166]. Doxorubicin-loaded DNA nanostructures were administered to regular human breast adenocarcinoma cancer cells. After one day of treatment, the drugs were co-localized in the cytoplasm and successfully induced cell death via confocal fluorescent analyses. Since DNA nanostructures are nontoxic and biocompatible, loaded drugs can be slowly released through the slow degradation of DNA nanostructures in vivo. In addition, peptides and antibodies can be integrated onto drug-loaded DNA nanostructures to improve the delivery activity and functionality of the delivery vehicles [166]. Applying DNA nanotechnology to drug delivery vehicles has not been explored extensively and this offers a great venue for next-generation drugs that are highly customizable and programmable.

### 6.2. DNA Nanostructures as Personalized Therapy

Personalized therapy proposes more specific, less invasive, treatments. The purpose of doing such is to target only unhealthy cells, which reduces toxicity and side-effects; thus, it needs specific biosensors for targeted biomarkers [127,168]. The use of DNA nanostructures for the detection and isolation of circulating tumor cells can help the identification of the type and source tissue of the cancer [169]. Overall, DNA nanostructures are potential tools for a more comprehensive specific diagnosis and treatment [170]. Novel biomarkers, such as Oncotype DX and MammaPrint, are complementing traditional ones—estrogen and progesterone receptors, and human epidermal growth factor receptor 2 (HER2)—as efficient prevention and prognostic tools for breast cancer [171]. Likewise, nanocarrier systems, such as polysorbates-coated NPs, nanocantilevers that deflect upon altered DNA proteins, and modified quantum dots, are implemented in different types of cancer therapies to trace the patient’s tumor molecular profile and improve intravascular drug delivery [172]. Functionalized AuNPs have become multifunctional devices that can be applied to genetic and protein biomarkers, which are combined with the molecular profile in order to detect cancerous cells [173]. 

## 7. DNA Amplification Systems

Upon detecting the presence of target biomarker, it is important to have a reliable and sensitive method to amplify the presence and signal of a target biomarker. Not only does DNA nanotechnology have the capability to drive the assembly of nanostructures, it also has the capability to amplify the signal of this molecule of interest both with and without the further need of enzymes. The following sections highlight a few robust DNA-based amplification systems that can be coupled with the prescribed biosensors for boosting signal sensitivity.

### 7.1. DNA Hybridization Chain Reaction (HCR)

**Figure 10:**
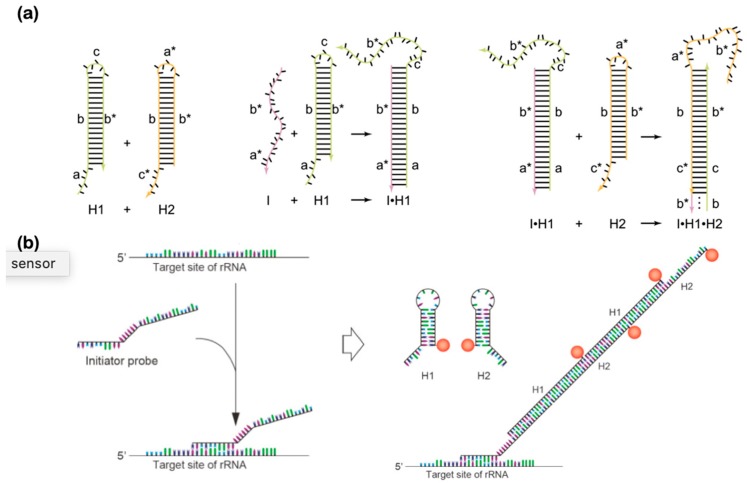
Principle of enzyme-free DNA hybridization chain reaction amplification: (**a**) Basis mechanism involved two programmable meta-stable DNA hairpins and a target initiator. Upon sensing the presence of the initiator, the hairpins polymerize and amplify the target for signal detection. Adopted with permission from [104]. Copyright 2004, PNAS. (**b**) Applied HCR to in-situ DNA-HCR for detecting rRNA. The initiator probe hybridizes to the target site of rRNA and triggers the HCR polymerization. Fluorescent-labeled hairpins in the polymerization products collectively emit significant fluorescence for detecting purposes. Adopted with permission from [174]. Copyright 2015, Wiley & Sons.

Unlike PCR which employs polymerases to amplify the target molecules, DNA HCR just relies on the programmability and self-assembly of DNA to amplify the target molecules isothermally [104]. A set of two distinct meta-stable DNA hairpins is designed to hybridize only in the presence of target molecules. Upon adding a trigger molecule such as DNA, RNA, enzyme, or protein of interest, the chain reaction automatically amplifies the signal via the readout of polymerization of the two meta-stable hairpins. The operations of HCR to transduce and amplify the signal are illustrated in Figure 10a. Two hairpins are inactive in the mixture until an initiator is introduced; it triggers polymerization of strands until the exhaustion of the hairpins. The amplification can be tuned by altering the size of either the stem or loop of each hairpin [104]. The utility of HCR has been adopted widely to detect relevant biomarkers. For instance, Figure 10b shows the hybridization process where the initiator is programmed so that it will partly hybridize upon detection of target rRNA, and the rest will trigger polymerization of the hairpins for amplification [174]. HCR is analogous to enzyme-based loop-mediated isothermal amplification and rolling circular amplification in the sense that the amplicon is a product of polymerization. Without requiring polymerases, DNA-based HCR-based amplifications are appealing to biosensing applications in which one can employ the isothermal sensing in remote inaccessible areas. 

### 7.2. DNA Catalytic Hairpin Assembly (CHA)

**Figure 11:**
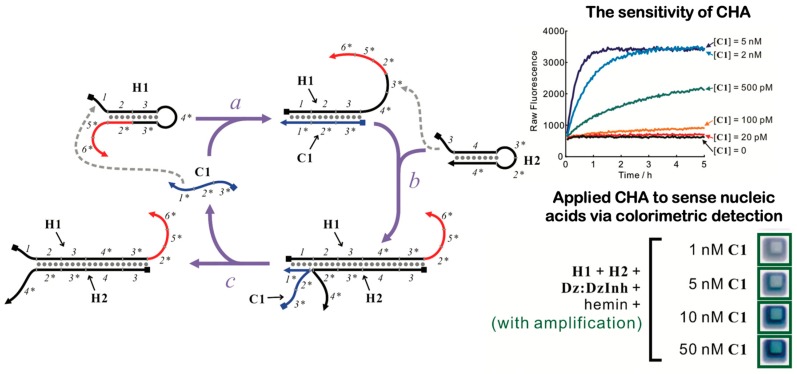
Principle of DNA catalytic hairpin assembly amplification (CHA): Basis mechanism involved two meta-stable DNA hairpins and a target initiator. Upon sensing the presence of the initiator, the hairpins autonomously cycle through the cascade reaction, producing exponential DNA output signals through either FRET or colorimetric analyses. Adopted with permission from [176]. Copyright 2011, Oxford University Press.

Similar to HCR, DNA CHA utilizes DNA’s programmability and self-assembly to amplify target molecules in the absence of polymerases [175]. The mechanism differs from HCR by re-using the meta-stable hairpin strands similar to the enzyme-free autocatalytic systems and does not require polymerization of strands as seen in the HCR approach. For instance, CHA relies on an initiator strand (C1) that catalyzes hybridization of the first hairpin (H1), and which then begins a cascade branch migration reaction measured by FRET (Figure 11) [176]. Each cycle produces a detectable DNA output and a re-usable catalyst for the next cycle. Unlike an entropy-driven autocatalytic system [102], CHA utilizes the secondary structure of the DNA hairpin, which allows for sequestration of active domains. Also unlike HCR systems [104], CHA amplifies the target by outputting the result to the secondary independent FRET reporter system. Since CHA and HCR offer a unique signal amplification function, a combined CHA-HCR system has recently been demonstrated to significantly boost the amplification factor [119]. In particular, a target biomarker, carcinoembryonic antigen (CEA), forms a double-stranded DNA and triggers hybridization of hairpins, creating copies that initiate amplification by HCR [177]. One of the main challenges of CHA reactions is to make their circuits thermostable, which require specific lengths of both hairpins and loops in order to ensure enough free energy and catalysis.

### 7.3. DNA Rolling Circle Amplification (RCA)

Combining PCR with circularized DNA templates, amplification here can be done by replicating circularized oligonucleotide probes under isothermal conditions [178,179,180], thus minimizing the need of thermal cycling. In particular, a template DNA is first ligated to form a circular structure. Upon detecting the target DNA strand, forming a double helix with the circularized DNA strand, the polymerase starts the amplification process by continuously extending the circularized DNA strand and making multiple-fold copies of the template until the resource is exhausted. Applied RCA has been used to detect mircoRNA, a small non-coding conserved RNA molecule which has important biological functions [178,181,182]. The potential amplification factors of each approach are obviously highly dependent on assay conditions and devices. Direct comparisons of the LOD among different systems can be misleading. However, enzyme-free approaches appear more attractive in an equipment-free environment, thus they can be quickly integrated and prototyped for next-generation point-of-care diagnostics.

## 8. DNA Kinesin-Like Motors

Utilizing DNA nanotechnology, the detection of target biomarkers and the amplification of target inputs with high sensitivity are important steps. However, actively searching and transporting DNA nanostructures in vivo are attractive features for next-generation autonomous systems. The following subsections highlight novel proof-of-concept DNA walkers and DNA motors which potentially can be integrated to the existing systems for advanced autonomous biosensing. 

### 8.1. DNA Walkers

DNA walkers are designed to operate autonomously. The design of such systems include sets of oligonucleotides functioning as the walker, the track, as well as the attachment and detachment fuel strands [120]. The track can be DNA nanostructures or other nanostructures including single-wall carbon nanotubes. The walker can be simply DNA sequences or DNA-labeled constructs including quantum dots, NPs, proteins, or enzymes [183,184,185]. DNA walkers are designed to be highly reliable, mobile, autonomous and controllable, in order to be used in applications such as transport [186]. Moreover, DNA walkers can work as signal amplification systems for electrochemical biosensors used in disease detection [187]. In particular, the walker is an aptamer and the track consists of DNA hairpins. Both aptamer and hairpins are immobilized on an electrochemical platform. Upon sensing a target protein (e.g., thrombin), a sandwich structure is formed through the binding of thrombin with the aptamer via a helper strand, forming a functional walker. The functional walker then hybridizes to a hairpin of the track and forms a Mg^2+^-dependent DNAzyme complex. In the presence of Mg^2+^, the complex is cleaved, releasing the functional walker to freely hybridize with another hairpin on the track and converting the hairpin into a G-quadruplex. Upon interacting with K^+^, the G-quadruplex binds to hemin. The functional walker continues traversing along the track, cleaving and converting hairpins to G-quadruplexes, resulting in significant electrochemical differences for signal amplification and detection. 

### 8.2. DNA Bipedal Motors

Similar to DNA walkers, DNA bipedal motors consist of a motor and a track. The only difference is that the motor has more than 1 foot to traverse along the track. When DNA bipedal motors walk over DNA tracks, they create a signal enhancement effect, which is the reason for incorporating DNA walker in biosensors [119]. However, one of the challenges in the design of autonomous DNA nanomotors is the synchronization of the DNA legs, so that more complex systems can be developed [188]. Additionally, a DNA origami track combined with a bipedal DNA walker is a viable alternative to other models, since it has shown high operational yield and speed [189]. To date, most DNA walkers and DNA bipedal motors have been demonstrated as a proof-of-concept. Due to the complexity of such systems, it remains relatively challenging to operate these systems in non-optimal conditions. However, it also presents a great opportunity to leverage such devices in next-generation DNA microsystems for biodiagnosis.

## 9. Discussion and Conclusions

The field of DNA nanotechnology has now been around for circa three decades. From a simple DNA junction to various kinds of DNA-based biosensors, the field has contributed a vast body of knowledge about controlling matter at the molecular scale. By simply utilizing intrinsic DNA self-assembly, DNA oligomers can be programmed to form predictable DNA nanostructures with high yield either through DNA tile assembly, DNA origami assembly, or DNA brick assembly. For applied biotechnological advances, synthesizing gram-scale levels of DNA nanostructures has been demonstrated to be feasible [190], thus scalability is possible especially if a large amount of a given DNA nanostructure is needed for a particular application. On the other hand, a small set of DNA oligomers can be programmed to form mechanical devices such as DNA tweezers, DNA walkers, or entropy-driven autocatalytic systems with highly predictable operations. In most biological applications, the stability of DNA systems has been found to remain relevant even in cell lysate in contrast to natural, single- and double-stranded DNA [191]. Indeed, the stability and functionality of DNA systems in various conditions (in vitro and in vivo) validate their use for next-generation biological applications although it is clear that much still remains to be learned on this critical subject [192,193].

The utility of DNA nanotechnology can also be extended to other disciplines. For instance, DNA systems can be synthesized with thiol, biotin, or appended peptides, or decorated with enzymes, proteins or fluorophores to greatly expand their functionalities; to some extent, this has also been illustrated in the above biosensing examples. Considerable work has also been undertaken in DNA systems that have been functionalized with different photoactive molecules for energy harvesting and transfer; this suggests that these systems should be amenable to complex types of information processing [183,194,195,196]. It is also important to note that as DNA systems get more complex and gain the capability of carrying many more and varied non-DNA molecules, the possibility exists that such systems could approach diminishing returns in which functionality is increased while the yield is decreased [197]. At the present time, most of the examples in the literature are primarily proof-of-concept demonstrations. This, in turn, suggests that it is important to now begin applying them with a strong emphasis on their reliability and reproducibility. Indeed, rigorous testing of DNA-based biosensors in different working conditions will be important to achieving reliable results as well as reproducibility for diagnostic, medical, and biosensing applications while still maintaining scalability and reliability [184,198]. Although substantial evidence of the intended functionality and safety for specific diagnostic situations can be achieved through reliability testing and reproducibility, there remain many other important factors for achieving regulatory approval when bringing DNA-based biosensors for use in the general public. The proposed DNA-based microsystems for biodiagnosis are not meant to be a total replacement to current medicine and diagnostics but a complementary approach to augment the current status quo as new technologies are developed.
